# Biodynamic signatures from ex vivo bone marrow aspirates are associated with chemotherapy‐induced neutropenia in cancer‐bearing dogs

**DOI:** 10.1002/vms3.423

**Published:** 2020-12-24

**Authors:** Blake A. Marcum, Zhe Li, John J. Turek, George E. Moore, David D. Nolte, Michael O. Childress

**Affiliations:** ^1^ Department of Veterinary Clinical Sciences College of Veterinary Medicine Purdue University West Lafayette IN USA; ^2^ Department of Physics and Astronomy College of Science Purdue University West Lafayette IN USA; ^3^ Department of Basic Medical Sciences College of Veterinary Medicine Purdue University West Lafayette IN USA; ^4^ Present address: Veterinary Specialty Center Buffalo Grove Illinois USA

**Keywords:** adverse events, cancer chemotherapy, precision medicine, spectroscopy

## Abstract

**Background:**

Neutropenia is the most common dose‐limiting side effect of cytotoxic chemotherapy in cancer‐bearing dogs. Biodynamic imaging (BDI) is a functional imaging technology that measures dynamic light scattering from living, three‐dimensional tissues to characterize intracellular motion within those tissues. Previous studies have associated BDI biomarkers with tumour sensitivity to chemotherapy agents in dogs with naturally occurring cancer. We hypothesized that BDI, performed ex vivo on bone marrow aspirate samples, would identify dynamic biomarkers associated with the occurrence of specific degrees of neutropenia in tumour‐bearing dogs receiving doxorubicin chemotherapy.

**Materials and Methods:**

Bone marrow aspirates were collected from 10 dogs with naturally occurring cancers prior to initiation of doxorubicin treatment. BDI was performed on bone marrow samples treated ex vivo with doxorubicin at 0.1, 1, 10 and 100 μM along with 0.1% DMSO as a control. Dogs then were treated with doxorubicin (30 mg/m^2^, intravenously). Peripheral blood neutrophil counts were obtained on the day of treatment and again 7 days later. Receiver operating characteristic curves identified provisional breakpoints for BDI biomarkers that correlated with specific changes in neutrophil counts between the two time points.

**Results:**

Provisional breakpoints for several BDI biomarkers were identified, specifying dogs with the largest proportionate change in neutrophils and with neutropenia that was grade 2 or higher following doxorubicin treatment.

**Conclusions:**

Biodynamic imaging of bone marrow aspirates may identify those dogs at greater risk for neutropenia following doxorubicin chemotherapy. This approach may be useful for pre‐emptively modifying chemotherapy dosing in dogs to avoid unacceptable side effects.

## Graphical abstract

Biodynamic imaging is a form of Doppler spectroscopy that records temporal fluctuations in back‐scattered light from living tissues as a surrogate measure of changes in intracellular motion within those tissues, which are induced by drug exposure. This drug response spectrogram illustrates a phenotypic, intracellular motion‐based response of canine bone marrow tissue to the cytotoxic agent, doxorubicin.

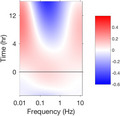

## 1 INTRODUCTION

The goal of cancer chemotherapy in companion animals is to extend survival time or improve quality of life without causing unacceptable side effects. Most cytotoxic chemotherapy drugs have a narrow therapeutic index, thus, the difference between an efficacious and a toxic dose may be slight. Myelosuppression, usually manifested by neutropenia, is the dose‐limiting side effect of most cancer chemotherapy drugs (Bisson et al., 2018; MacDonald, 2009). Some degree of neutropenia is inevitable following myelosuppressive chemotherapy, and may be associated with a positive response to treatment (Sorenmo, Overly, et al., 2010; Wang et al., 2015) However, neutropenia also may lead to treatment delays and chemotherapy drug dose reductions, both of which may be deleterious to patient survival (Kuderer et al., 2006; Lyman, 2009; Vail, 2009) Moderate‐to‐severe neutropenia (≤1,000 μL^−1^) predisposes patients to bacterial sepsis that may be costly to treat, and is associated with increased morbidity and mortality (Kuderer et al., 2006; Vail, 2009).

Identified risk factors for neutropenia in dogs receiving chemotherapy include receiving treatment in the early portions of a chemotherapy protocol or receiving treatment with specific drugs, such as doxorubicin or vincristine (Sorenmo, Harwood, et al., 2010). Dogs harbouring the ABCB1‐1Δ mutation in the *MDR1* gene also have been shown to be at greater risk for experiencing neutropenia following vincristine treatment (Mealey et al., 2008). However, a means to reliably and accurately predict treatment‐related neutropenia in individual dogs remains elusive because each dog's tolerance for chemotherapy may be affected by many factors, such as interpatient variability in drug pharmacokinetics (PKs) and the inherent sensitivity of an individual dog's hematopoietic cells to injury by a given drug dose (Boria et al., 2004; Undevia et al., 2005). Therefore, a tool to predict neutropenia prior to treatment would be valuable for identifying those dogs for which prophylactic measures (e.g. antimicrobial therapy) should be considered to prevent serious treatment‐related morbidity.

Biodynamic imaging (BDI) is a new form of ex vivo functional imaging microscopy with potential to address this need. BDI uses intracellular Doppler spectroscopy to measure dynamic low‐intensity light scattering from living tissues to generate dynamic biomarkers of intracellular motion. BDI characterizes phenotypic responses of three‐dimensional tumour tissue biopsy samples to cytotoxic chemotherapy drugs in the ex vivo setting, associating these responses to the in vivo clinical response of the parent tumour to those drugs (An et al., 2013; Choi et al., 2018; Custead et al., 2015). The technique profiles living, three‐dimensional tissues within a natural extracellular matrix rather than an artificial two‐dimensional cell culture system, allowing the characterization of unique tissue responses to a variety of cancer chemotherapy drugs (Jeong et al., 2007; Nolte et al., 2012). Intracellular motion data captured by BDI are displayed as drug response spectrograms, which are time‐frequency representations depicting changes in intracellular motion across a range of frequencies over time. These spectrograms characterize changes in physiology across several cellular compartments, from organelle motion to motion affecting the whole cell, revealing a tissue's unique phenotypic response to a particular drug (Custead et al., 2015; Li et al., 2019; Nolte et al., 2011, 2012).

Previous work in tumour‐bearing dogs has shown BDI to be potentially predictive of tumour response to cytotoxic drug treatment. In one study, BDI was used to image ex vivo tumour biopsy samples from dogs with naturally occurring, treatment‐naïve non‐Hodgkin lymphoma. A single motion‐based biomarker recorded by BDI showed perfect (100%) correlation with objective tumour response to doxorubicin in vivo (Custead et al., 2015). In a follow‐up study in dogs with diffuse large B‐cell lymphoma receiving CHOP chemotherapy, BDI performed with 84% accuracy in associating dynamic biomarkers from ex vivo tumour tissue samples to the long‐term response of the dogs’ cancers to treatment (Choi et al., 2018) Although BDI has not previously been used to characterize drug responses in non‐neoplastic tissues, it is reasonable to speculate that BDI may be useful for predicting in vivo treatment‐related adverse events when performed ex vivo on samples from normal tissues. We hypothesized that BDI, performed ex vivo on bone marrow aspirate samples, would identify intracellular motion‐based biomarkers correlating with the occurrence of specific degrees of neutropenia in cancer‐bearing dogs receiving doxorubicin.

## 2 MATERIALS AND METHODS

### 2.1 Enrolment

This was a limited‐scale feasibility study intended to establish possible correlations between BDI data and the occurrence of neutropenia in dogs receiving chemotherapy. The planned study population was 10 client‐owned dogs. Dogs were eligible for the study if they had a histologically confirmed cancer that would be treatable with doxorubicin. A standard eligibility screening protocol was used to evaluate general health and cancer stage in all dogs. Staging tests included complete blood count (CBC), serum biochemistry, bone marrow aspirate cytology, electrocardiogram, thoracic radiography and abdominal ultrasonography. Dogs weighing <15 kg, with cardiac disease reasonably precluding doxorubicin treatment, or with breed‐associated risk for carrying the ABCB1‐1Δ mutation (Gustafson & Thamm, 2010; Mealey & Meurs, 2008) were excluded from the study. Dogs also were excluded if they had evidence of pre‐existing bone marrow dysfunction, defined as peripheral cytopenias (e.g. haematocrit <37.0%, neutrophils <3,000 μL^−1^ or platelets <200,000 μL^−1^) or neoplastic bone marrow infiltration observed on cytological review of aspirates. Dogs that had received prior chemotherapy (including glucocorticoids in dogs with lymphoma) or radiotherapy were excluded due to possible alterations in bone marrow physiology elicited by these treatments, which may have affected the in vivo or ex vivo bone marrow response to doxorubicin. Finally, although an effect of hepatic dysfunction on doxorubicin PKs and bone marrow exposure to doxorubicin is inconsistently documented in humans, (Ackland et al., 1989; Twelves et al., 1998) dogs with increased serum liver enzyme activity or serum bilirubin concentration were excluded to limit potential sources of confounding in the interpretation of study results.

### 2.2 Bone marrow collection and handling

All dogs were sedated with dexmedetomidine (5–10 μg/kg IV) and butorphanol (0.1–0.2 mg/kg IV) for bone marrow sampling. Bone marrow aspirates were collected from the left or right proximal humerus using a 13g Illinois sternal biopsy needle. A 2 ml marrow sample was aspirated into a 12 ml syringe pre‐loaded with 1 ml EDTA. Approximately 0.5–1.0 ml of the aspirate was submitted for cytological review as part of the eligibility screening described above. The remaining portion was transferred into a 15 ml conical tube containing 5 ml of RPMI‐1640, then immediately transported to a nearby laboratory where BDI was performed on the same day. Macroscopic marrow particles (approximately 0.5–1 mm diameter) were placed in 96 well plates and immobilized in low‐gel temperature porous agarose for BDI as previously described (An et al., 2013; Choi et al., 2018; Custead et al., 2015; Jeong et al., 2007; Li et al., 2019; Nolte et al., 2011, 2012). Bone marrow samples were treated with doxorubicin (SelleckChem) at 0.1, 1, 10 and 100 μM, along with 0.1% DMSO as a control. Plasma concentrations of 0.1 and 1.0 μM are achievable following administration of clinically relevant doses of doxorubicin to cancer‐bearing dogs, while 10 and 100 μM are supraphysiologic (Gustafson et al., 2002; Wilke et al., 1991; Wittenburg et al., 2019).

### 2.3 Biodynamic imaging

**FIGURE 1:**
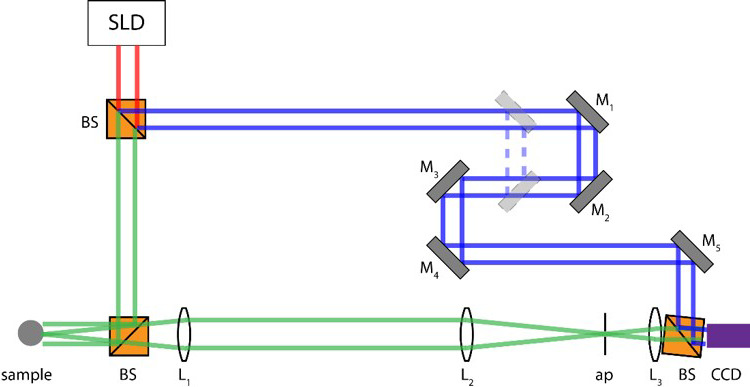
The biodynamic imaging system. The BDI instrument is designed in the configuration of a Mach‐Zehnder interferometer, which creates off‐axis holograms of the object being imaged. The light source for the system is a superluminescent diode (SLD), with a centre wavelength at 836.2 nm and a maximum power of 22.9 mW. The signal arm of the light beam (green lines) projects downward to illuminate the target tissue sample, while the reference arm (blue lines) projects to the right of the schematic. Back‐scattered light from the target tissue interferes with the reference arm to create a hologram that is captured on a CCD camera mounted at the Fourier plane of the imaging system. Mirrors (M_1_ and M_2_) mounted within the translational stage can be moved laterally to the left and right, changing the focal depth within the target tissue. This allows a series of holograms to be collected in tomographic fashion across a range of depths from tissues up to 1 mm thick. ap, aperture; BS, beam splitter, L_1_‐L_3_, lenses; M_1_‐M_5_, mirrors

**FIGURE 2:**
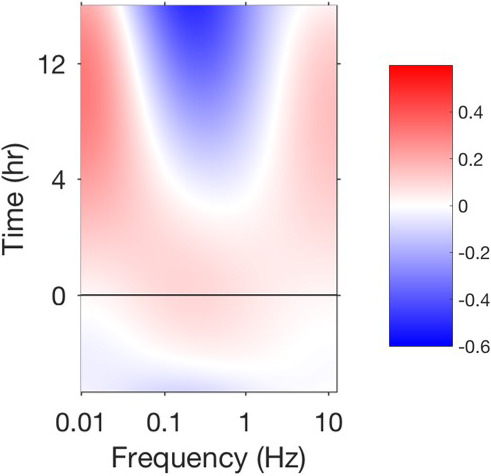
Drug response spectrogram produced by biodynamic imaging following application of doxorubicin to bone marrow tissue from a cancer‐bearing dog. Drug response spectrograms are time (*y*‐axis) versus frequency (*x*‐axis) representations of intracellular motion occurring across a frequency range 0.01–12.5 Hz (3 nm/s to 4 μm/s). This frequency range describes motions of varying scales. Signals in the low‐frequency range (0.01–0.1 Hz) are associated with large‐scale motions, such as changes in the shape or integrity of the entire cell. Smaller‐scale motions, such as membrane undulations or nuclear motions, are recorded in the mid‐frequency range (0.1–0.5 Hz). The smallest‐scale motions, such as those associated with organelle and vesicle transport, are recorded at the highest frequencies (0.5–12.5 Hz). The false colour scale bar to the right of the image denotes regions of the spectrogram where motion increases (red) or decreases (blue) as a function of time. The black horizontal line at time = 0 denotes the time at which doxorubicin was applied to the tissue. This spectrogram depicts a phenotypic response in which both low‐ and high‐frequency motions within the tissue increase following doxorubicin application, while mid‐frequency motion decreases. This phenotypic response has been shown previously to correlate with apoptosis experienced by cells within the imaged tissue (Nolte et al., 2012)

The BDI instrument (Figure 1) records temporal fluctuations in low‐coherence light back‐scattered through immobilized tumour tissue samples. The intensity fluctuations of this back‐scattered light are a surrogate measure of intracellular motion within those tissues through the interference of multiply scattered Doppler frequencies associated with intracellular motion (Li et al., 2019). Biodynamic measurements of intracellular motion can be taken in tomographic fashion at several depths, but typically are acquired as an “optical section” at a fixed light‐path depth, usually about 0.4 mm deep inside the biopsy tissue samples. BDI data are presented visually as drug response spectrograms (Figure 2), which are time versus frequency representations of relative changes in intracellular motion occurring across a range of frequencies, both prior to and following ex vivo drug treatment (An et al., 2013; Choi et al., 2018; Custead et al., 2015; Jeong et al., 2007; Li et al., 2019; Nolte et al., 2011, 2012). The frequency range captured by BDI spans three orders of magnitude, from 0.01 to 12.5 Hz for Doppler frequencies associated with speeds of 3 nm/s to 4 μm/s respectively.

The extent to which specific patterns of change in Doppler frequencies associated with intracellular motion occur in a tissue following drug treatment is quantifiable with BDI. These quantities are recorded as biomarker values, which can be submitted to statistical analysis to identify patterns of intracellular motion associated with clinically meaningful phenotypic responses of a tissue to a drug. Approximately 40 different BDI biomarkers have been described to date, (An et al., 2013; Choi et al., 2018; Custead et al., 2015; Jeong et al., 2007; Li et al., 2019; Nolte et al., 2011, 2012) although not all of these provide clinically relevant information about the phenotypic response of a given tissue to a given drug.

The biomarkers recorded by BDI reflect specific patterns of cell motion either occurring *globally* across the entire spectrogram or occurring only *locally*, within confined time‐frequency regions of the spectrogram. The magnitude of the biomarker values is reported in relative units (RU), which describe the extent to which drug treatment elicits a change in a specific pattern of intracellular motion (i.e. a biomarker value) relative to baseline motion measurements. RU values are specific to a given biomarker, thus values of the same magnitude for two different biomarkers do not reflect an identical magnitude of effect. Biomarker values can be either positive or negative, depending upon whether a given motion pattern became more pronounced or less pronounced following drug treatment.

In the present study, BDI measurements were obtained from bone marrow samples from all dogs. The BDI system measured 4 hr of baseline behaviour prior to ex vivo doxorubicin treatment, then all bone marrow samples were treated at the specified concentrations of doxorubicin, remaining at this fixed level of drug exposure while BDI measurements were obtained continuously for 10 hr, as previously described (Choi et al., 2018). Imaging was performed on four tissue replicates at each doxorubicin concentration from each dog, and averaged (mean) biomarker values across these four replicates were submitted for subsequent statistical analysis.

### 2.4 Medical management of dogs

In all dogs, a 3 ml blood sample was collected in EDTA for a pre‐treatment CBC at the time of bone marrow collection. All dogs received doxorubicin at 30 mg/m^2^ intravenously on the same day as blood and bone marrow collection. Following doxorubicin treatment, all dogs were prescribed tramadol to be given orally at a dose of approximately 5 mg/kg every 8 hr for approximately 3 days to provide analgesia after bone marrow sampling. A CBC was repeated 7 days later, at the time of the expected neutrophil nadir. Neutropenia was defined as an absolute neutrophil count <3,000 μL^−1^, the low end of the normal reference range for peripheral blood neutrophil counts in dogs at our institution's clinical pathology laboratory. Neutropenia was graded according to the Veterinary Cooperative Oncology Group‐Common Terminology Criteria for Adverse Events (VCOG‐CTCAE 1.1) (Veterinary Cooperative Oncology Group, 2016) The pre‐ and post‐doxorubicin neutrophil counts, as well as the absolute and proportionate difference in these values, were recorded.

### 2.5 Statistical analysis

BDI biomarkers associated with Doppler frequency shifts likely to have high biological significance were selected to test for statistical associations with clinical outcomes, as previously described (Choi et al., 2018; Nolte et al., 2012). The process of biomarker selection for data analysis is an iterative one that begins with comparison of each drug response biomarker to the average of all biomarkers, including the DMSO control. Biomarkers showing closely associated features are pooled to improve their signal‐to‐noise ratio during this initial process. After pooling is completed, both the pooled and raw biomarker data are normalized with respect to the smallest standard deviation of a biomarker set of common origin within the spectrogram (i.e. local biomarkers are normalized with respect to the smallest standard deviation of a set of local biomarkers, global biomarkers normalized with respect to the smallest standard deviation of a set of global biomarkers and so forth). Following normalization, the effect of each raw or pooled biomarker is determined by calculating a *z*‐factor. Biomarkers with the greatest *z*‐factors are tabulated, then iteratively analysed with respect to effect on a clinical outcome in a one‐hold‐out fashion until those biomarkers with consistently high *z*‐factor values are identified.

Once biomarkers with high *z*‐factor values were selected for the present study, scatter plots were constructed to identify the subset of these biomarkers appearing to have good ability to discriminate potentially clinically meaningful outcomes related to neutropenia. Specifically, these outcomes included the absolute and proportionate changes in neutrophil counts, as well as VCOG grade of neutropenia. The numerical values for these biomarkers were analysed using receiver operating characteristic (ROC) curves to identify provisional cut‐points that would discriminate dogs with specific degrees of neutropenia. The accuracy of each selected biomarker for identifying specific degrees of neutropenia was determined by calculating its area under the ROC curve with its binomial exact 95% confidence interval.

## 3 RESULTS

Ten dogs were enrolled in the study. Seven dogs had multicentric lymphoma, two dogs had hemangiosarcoma and one dog had osteosarcoma. An accurate pre‐treatment platelet count could not be obtained from one dog with lymphoma due to platelet clumping. However, this dog's platelet count was normal (287,000 μL^−1^) on a CBC performed by its primary care veterinarian 14 days prior to presentation, and its bone marrow aspirate cytology was unremarkable, with adequate numbers of megakaryocytes noted. It was therefore decided to include the dog in the study. Data describing the pre‐ and post‐treatment neutrophil counts for all 10 dogs are summarized in Table 1. Three dogs did not become neutropenic after treatment with doxorubicin. Four dogs experienced grade I neutropenia (<3,000 μL^−1^ to 1,500 μL^−1^), one dog experienced grade II neutropenia (<1,499 μL^−1^ to 1,000 μL^−1^) and two dogs experienced grade III neutropenia (<999 μL^−1^ to 500 μL^−1^).

**TABLE 1:** Data describing occurrence and severity of neutropenia in 10 dogs receiving single‐agent doxorubicin chemotherapy

Dog	Tumour type	Pre segs (10^3^/μl)	Post segs (10^3^/μl)	Absolute Δ (10^3^/μl)	Proportionate Δ	Grade
1	LSA	7.6	3.8	−3.8	−0.500	0
2	LSA	10.1	4.2	−5.9	−0.584	0
3	LSA	30.0	0.8	−29.2	−0.973	3
4	HSA	10.7	2.0	−8.7	−0.813	1
5	LSA	8.2	1.6	−6.6	−0.805	1
6	HSA	5.0	2.5	−2.5	−0.500	1
7	LSA	12.6	2.4	−10.2	−0.810	1
8	LSA	4.2	1.4	−2.8	−0.667	2
9	OSA	5.6	3.3	−2.3	−0.411	0
10	LSA	10.2	0.7	−9.5	−0.931	3

Abbreviations: Absolute Δ, post segs−pre segs; Grade, grade of neutropenia according to the Veterinary Cooperative Oncology Group Common Terminology Criteria for Adverse Events v1.1 (Veterinary Cooperative Oncology Group, 2016); HSA, hemangiosarcoma; LSA, lymphoma; OSA, osteosarcoma; Post segs, segmented neutrophil count on Day 8; Pre segs, segmented neutrophil count on Day 1; Proportionate Δ, (post segs−pre segs)/pre segs.

**FIGURE 3:**
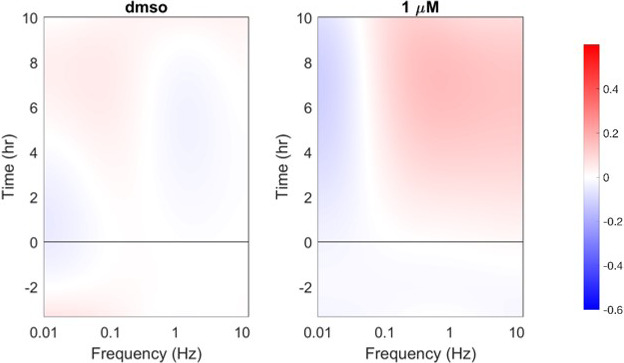
Drug response spectrograms illustrating the derivation of one of the biomarkers of apparent clinical significance in this study, *Hi Dox 1/CTRL*. This is a local biomarker, reflecting changes in intracellular motion occurring only in the high‐frequency band of the spectrogram. The biomarker reflects the change in high‐frequency motion in bone marrow samples treated at 1 μM doxorubicin relative to DMSO control. The spectrograms illustrate a phenotypic response of canine bone marrow to doxorubicin characterized by a large positive *Hi Dox 1/CTRL* value. This is based on the increased high‐frequency motion in the sample treated at 1 μM relative to the control sample. Numerical values for the *Hi Dox 10/1* biomarker are similarly derived, reflecting the change in high‐frequency motion in bone marrow samples treated at 10 μM doxorubicin relative to 1 μM (not shown in figure)

**FIGURE 4:**
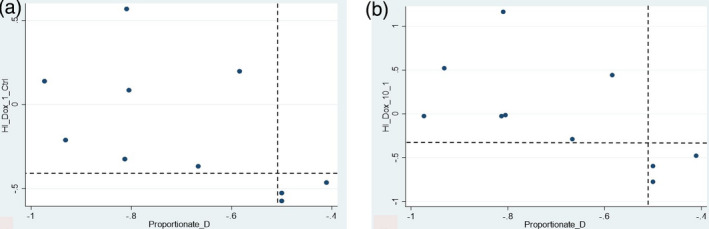
Scatter plots depicting provisional cut‐points for BDI biomarkers *Hi Dox 1/CTRL* (a) and *Hi Dox 10/1* (b) as predictors of proportionate change in neutrophils. Biomarker values are plotted on the *y*‐axis, and proportionate change in neutrophils is plotted on the *x*‐axis. The dashed horizontal lines represent provisional cut‐points that discriminated dogs with >50% proportionate decrease in their neutrophil counts from those with ≤50% proportionate decrease

Thirteen biomarkers with sufficiently high *z*‐factors were examined for associations with clinical outcomes related to neutropenia using scatter plots. Four biomarkers showed promising ability to discriminate those dogs with greater proportionate degrees of neutropenia. Two such biomarkers—*Hi Dox 1/CTRL* and *Hi Dox 10/1*—describe the change in motion at the high frequency range of the spectrogram (caused by alterations in organelle motions) seen at doxorubicin 1 μM relative to control, and at doxorubicin 10 μM relative to 1 μM respectively (Figure 3). Provisional cut‐points for these two biomarkers were identified, discriminating with 100% accuracy (95% CI 69.2%–100%; Table 2) those dogs with >50% decrease in their neutrophil counts from those with lesser proportionate change (Figure 4).

**TABLE 2:** Areas under the receiver operating characteristic curves (AUC) for four selected BDI biomarkers, with corresponding exact binomial 95% confidence intervals (CI)

Biomarker	AUC	95% CI	Optimal cutoff	TP% (Sn)	FP% (1‐Sp)
*Hi Dox 1/Ctrl*	1.000	0.692–1.000	≥ −0.366	100%	0%
*Hi Dox 10/1*	1.000	0.692–1.000	≥ −0.288	100%	0%
*DNSD Dox 10/CTRL*	0.857	0.555–0.997	≥0.263	100%	14.29%
*DNSD Dox 10/1*	0.810	0.444–0.975	≥0.160	100%	28.57%

Note

From the ROC curve analysis, optimal cutoff values were identified for each biomarker, along with their associated true‐positive (TP) and false‐positive (FP) rates. The values for the *Hi Dox 1/CTRL* and *Hi Dox 10/1* biomarkers describe their accuracy for discriminating dogs with a proportionate decrease in circulating neutrophil count of >50% from those with a decrease of ≤50%. Values for the *DNSD Dox 10/CTRL* and *DNSD Dox 10/1* biomarkers describe their accuracy for discriminating dogs with VCOG grade ≥2 neutropenia from those with VCOG grade <2.

Abbreviations: Sn, sensitivity; Sp, specificity.

**FIGURE 5:**
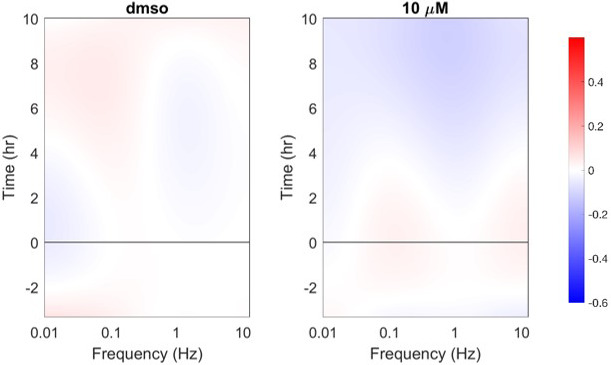
Drug response spectrograms illustrating the derivation of the *DNSD Dox 10/CTRL* biomarker. The normalized standard deviation (NSD) of each pixel in a drug response spectrogram is defined as the standard deviation of the intensity fluctuations of each pixel divided its average intensity. NSD is a global biomarker, reflecting aggregate intracellular motion occurring across the full frequency range of the spectrogram. DNSD (or ΔNSD) simply reflects the change in aggregate intracellular motion (NSD) across all pixels in the spectrogram as a function of time. These spectrograms illustrate a phenotypic response of canine bone marrow to doxorubicin characterized by a large negative *DNSD Dox 10/CTRL* value. This is based on the decreased motion across all frequencies in the sample treated at 10 μM relative to the control sample

**FIGURE 6:**
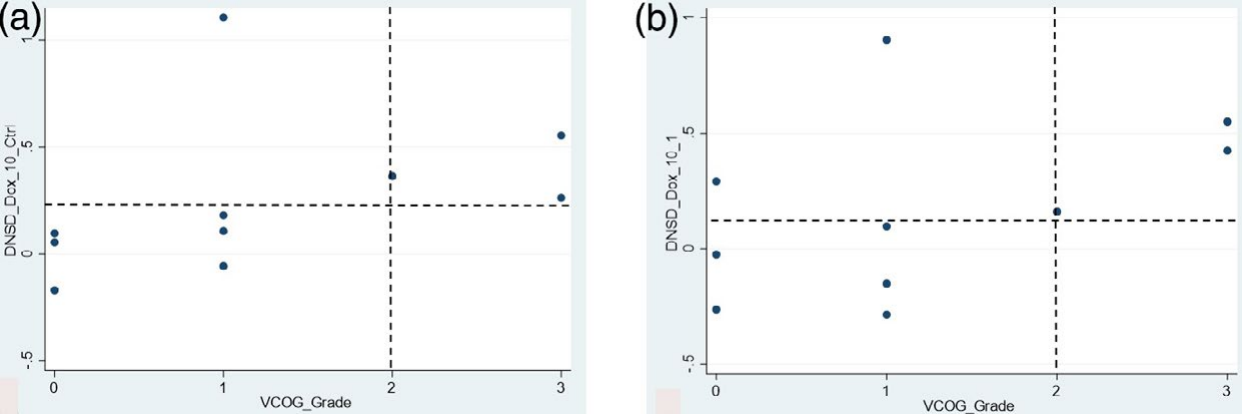
Scatter plots depicting provisional cut‐points for BDI biomarkers *DNSD Dox 10/CTRL* (a) and *DNSD Dox 10/1* (b) as predictors of VCOG grade of neutropenia. Biomarker values are plotted on the *y*‐axis, and VCOG grade is plotted on the *x*‐axis. The dashed horizontal lines represent provisional cut‐points that discriminated dogs with neutropenia of VCOG grade ≥2 from those with lower‐grade neutropenia. Note that the cut‐point for *DNSD Dox 10/CTRL* misclassified 1 dog (upper left quadrant, Figure 6a), while that for *DNSD Dox 10/1* misclassified 2 dogs (upper left quadrant Figure 6b)

An additional biomarker that showed promise as a predictor of potentially significant neutropenia was *DNSD* (or *ΔNSD*). This biomarker describes changes in aggregate motion over time across all frequencies of the spectrogram when compared with baseline values (Figure 5). Greater (more positive) *DNSD* values are associated with increased aggregate motion across all measured frequencies, while lesser (more negative) *DNSD* values are associated with decreased aggregate motion. The *DNSD Dox 10/CTRL* and *DNSD Dox 10/1* biomarkers reflect the change in aggregate intracellular motion (*DNSD*) in bone marrow samples treated with doxorubicin 10 μM relative to control and 10 μM relative to 1 μM, respectively. When comparing these values with VCOG grade, provisional cut‐points correlating with clinical outcomes of potential significance were again identified (Figure 6). Specifically, greater values of both biomarkers were associated with higher VCOG grades of neutropenia. Provisional cut‐points for *DNSD Dox 10/1* correctly identified dogs with neutropenia of VCOG grade ≥2 in 8/10 cases (accuracy 80%, 95% CI 55.9%–99.7%; Table 2), and for *DNSD Dox 10/CTRL* in 9/10 cases (accuracy 90%, 95% CI 44.4%–97.5%; Table 2).

## 4 DISCUSSION

Ex vivo BDI previously was shown to be potentially predictive of in vivo tumour sensitivity to chemotherapy in dogs with lymphoma (Choi et al., 2018; Custead et al., 2015). The study presented here suggests that BDI also may be useful for predicting the response of normal (non‐neoplastic) tissues, such as bone marrow, to chemotherapy. When comparing BDI data with clinical results, we identified provisional cut‐points for four BDI biomarkers that correlated with greater proportionate decreases in neutrophil counts or higher VCOG grades of neutropenia in dogs receiving doxorubicin chemotherapy. These preliminary results suggest that performing BDI on bone marrow samples prior to chemotherapy may provide clinicians with information about a patient's likely tolerance for a given drug, allowing decisions for dose modification to be made prior to treatment. Although the results in this small population of dogs require confirmation in larger trials, they suggest an interesting new application of BDI for the individualization of cancer chemotherapy dosing.

The utility of BDI for guiding chemotherapy drug dose adjustment was suggested in two ways by our results. First, two BDI biomarkers, *Hi Dox 1/CTRL* and *Hi Dox 10/1*, were able to accurately classify all 10 dogs based on a proportionate decrease in their neutrophil count by >50% following doxorubicin treatment. Although there was wide variability in the values for these two biomarkers, all three dogs whose neutrophil counts reduced by ≤50% had low (more negative) values of both biomarkers (Figure 4). Greater (more positive) values of these two *Hi Dox* biomarkers are associated with increased organelle and vesicle traffic, (Nolte et al., 2011, 2012) which is characteristic of drug‐induced cell death. This would be an undesired result in the bone marrow of a cancer patient. In contrast, dogs that have lesser values of the *Hi Dox* biomarkers may be less likely to undergo bone marrow cell death, suggesting a resilience to treatment. This suggests that low (more negative) values for these two biomarkers may be useful for identifying dogs that are reasonable candidates for chemotherapy dose escalation as a means to improve the anti‐cancer efficacy of treatment.

Second, two additional BDI biomarkers also appeared to correlate with VCOG grade of neutropenia. The biomarkers *DNSD Dox 10/CTRL* and *DNSD Dox 10/1* were both able to discriminate the majority of dogs with VCOG grade 2 or 3 neutropenia from those with lower‐grade neutropenia, although neither biomarker had a perfect correlation with clinical outcome (Figure 6). Greater (more positive) *DNSD* values are consistent with greater *Hi Dox* values, both reflecting activated cellular processes associated with drug insult (Nolte et al., 2011, 2012) and a greater degree of cellular injury, thus predictably correlating with greater degrees of neutropenia. Although many dogs tolerate grades 2 and 3 neutropenia without clinical evidence of illness, neutropenia of this degree may necessitate chemotherapy dose reductions and/or dose delays. The resulting alterations in chemotherapy dosing schedules are both inconvenient to pet owners and potentially deleterious to cancer‐bearing dogs, as they may reduce the overall dose intensity of a chemotherapy protocol and thereby compromise the protocol's efficacy. Future studies could help to identify more accurate breakpoints for these two BDI biomarkers so that dogs at risk for clinically significant complications of chemotherapy, such as febrile neutropenia or sepsis, may be accurately identified before treatment is given. This may facilitate early dose adjustment to enable better adherence to a chemotherapy dosing schedule, or identification of dogs that would benefit from prophylactic chemotherapy dose reduction or supportive care measures (e.g. antibiotics).

Several other techniques have been evaluated for predicting chemotherapy‐induced neutropenia in either dogs or humans with cancer. PK data have been used to construct models predictive of neutropenia in both dogs and humans receiving doxorubicin (Ackland et al., 1989; Wittenburg et al., 2019). More complex pharmacokinetic‐pharmacodynamic (PKPD) modelling strategies for predicting neutropenia also have been proposed (Wallin et al., 2009). Both PK and PKPD modelling strategies can be used to guide chemotherapy dose modification to avoid serious neutropenia. A disadvantage to these strategies is that they cannot be used a priori to select a starting drug dose for an individual patient—they require input data related to the administration of at least one drug dose. A priori drug dose selection can, in theory, be guided by clonogenic assays performed on bone marrow progenitor cells harvested from patients prior to drug treatment (Moneta et al., 2003; Parchment et al., 1998) Non‐clonogenic in vitro assays of drug cytotoxicity to hematopoietic progenitor cells also have been described (Haglund et al., 2010). Disadvantages to these clonogenic and non‐clonogenic cytotoxicity assays include technical complexity and a relatively long test turnaround time (7–14 days).

By comparison, BDI may allow for a priori prediction of chemotherapy‐induced myelosuppression using a technically simple test with rapid turnaround time (24 hr). The mechanical origins of Doppler light scattering from living tissue are based on the direct connection of Doppler light scattering to intracellular processes (i.e. metabolism, proliferation, cell death). However, while the biological relevance of some spectral patterns resolved by BDI has been determined (Figure 2), (Nolte et al., 2012) the precise changes in cell physiology associated with most BDI biomarkers have not yet been identified. Mapping the connections between the physical data provided by BDI with biological data—such as changes in gene expression, protein function or cellular metabolism—elicited by drug treatment is a crucial next step to more fully implementing BDI as a tool for predictive medicine in a clinical setting.

This study has a number of limitations. As this was a feasibility study, only a small number of dogs was enrolled. This limited our ability to comprehensively analyse the full spectrum of the data we collected. BDI records the phenotypic responses of living tissues to drugs by mapping intracellular motion occurring over three orders of magnitude on the frequency scale, generating datasets of the same scope as whole genome‐ or RNA‐sequencing experiments for each tissue sample analysed. To interpret the data with greater granularity, we collapsed it into discrete information channels (biomarkers) reflecting very specific patterns of cell motion on the time‐frequency spectrogram. In so doing, it is possible that we disregarded intracellular motion patterns containing clinically important data. Although our preliminary results here suggest that some BDI biomarkers may be useful in developing predictive models of chemotherapy‐induced myelosuppression, larger studies involving a greater number of dogs will be needed to identify the biomarker or combination of biomarkers with the greatest capacity to predict specific degrees of neutropenia.

Another limitation to this study was that few high‐grade adverse events occurred. Two of ten dogs (20%) experienced grade III neutropenia, but as no episodes of grade IV (i.e. potentially life‐threatening) neutropenia occurred, it is unknown how well BDI data would correlate with such events. Additionally, while it is common practice to obtain a post‐doxorubicin nadir neutrophil count 7 days following treatment, the nadir point likely varies from dog to dog. Thus, it is conceivable that we did not record the true neutrophil nadir in all dogs, which in turn may have led to misclassification of outcomes related to neutropenia.

Finally, we recognize that interpatient variability in drug PKs, which affects bone marrow exposure to doxorubicin, plays a major role in determining the risk for neutropenia in individual dogs. This effect is likely independent of the bone marrow's inherent sensitivity to the cytotoxic effects of doxorubicin. In this study, we did not assess in vivo plasma PKs of doxorubicin or estimate bone marrow exposure to this drug. A recent study in dogs receiving doxorubicin showed that the area under the curve for doxorubicin plasma concentration versus time was correlated with the circulating neutrophil count at the anticipated nadir point (Wittenburg et al., 2019). Although each dog in the current study was treated with the same doxorubicin dose (30 mg/m^2^), and we attempted to exclude dogs with organ dysfunction that might alter drug disposition, doxorubicin exposure to the bone marrow has been shown to be highly variable even among tumour‐bearing dogs of good general health status (Wittenburg et al., 2019). This variability may have affected outcomes related to neutropenia in a way that confounded our interpretation of the BDI data. Future attempts at predicting chemotherapy‐induced myelosuppression using BDI should therefore include plasma and/or tissue PK studies to determine the extent to which drug disposition and BDI data are correlated.

## 5 CONCLUSION

This study demonstrated that BDI, performed ex vivo on bone marrow tissues from chemotherapy‐treated dogs, shows promise as a clinical test that could predict neutropenia induced by doxorubicin treatment. Four BDI biomarkers correlated well with outcomes related to neutropenia, including proportionate decrease in circulating neutrophils and VCOG grade of neutropenia at the expected nadir point. The extent to which these, and other, BDI biomarkers can form a reliable predictive model for chemotherapy‐induced neutropenia must be tested in larger‐scale studies that also incorporate variables of demonstrated relevance to this outcome, such as PK data. Validating the biological mechanisms underlying the Doppler spectral data resolved using BDI will further strengthen continued predictive model development.

## CONFLICTS OF INTEREST

D. Nolte and J. Turek have a financial interest in Animated Dynamics, Inc. which has licensed biodynamic imaging technology for commercial development from the Office of Technology Commercialization at Purdue University.

## ETHICS STATEMENT

The authors confirm that the ethical policies of the journal, as noted on the journal's author guidelines page, have been adhered to and the appropriate ethical review committee approval has been received. Specifically, the study protocol pertaining to the animals used in this work was reviewed and approved by both the Purdue Animal Care and Use Committee (PACUC) and Veterinary Clinical Studies Committee (which serves in a capacity similar to that of an Institutional Review Board for trials involving human subjects). The approved PACUC protocol number was 1701001526. Written, informed consent was obtained from the owner of each dog before enrolling it in this study.

## AUTHOR CONTRIBUTION

**Blake Marcum:** Funding acquisition; Investigation; Project administration; Writing‐original draft. **Zhe Li:** Formal analysis; Investigation; Methodology; Writing‐original draft. **John Turek:** Investigation; Methodology; Writing‐original draft. **George E Moore:** Data curation; Formal analysis; Writing‐original draft. **David Nolte:** Conceptualization; Data curation; Formal analysis; Investigation; Methodology; Supervision; Validation; Writing‐review & editing. **Michael Childress:** Conceptualization; Data curation; Funding acquisition; Investigation; Methodology; Project administration; Resources; Supervision; Writing‐original draft; Writing‐review & editing.

### Peer Review

The peer review history for this article is available at https://publons.com/publon/10.1002/vms3.423.
